# Targeting fat browning in hypermetabolic conditions: a clinical perspective

**DOI:** 10.2144/fsoa-2019-0139

**Published:** 2020-01-21

**Authors:** Supreet Kaur, Priyal Shah, Marc G Jeschke

**Affiliations:** 1 Department of Biological Sciences, Ross Tilley Burn Centre, Sunnybrook Health Sciences Centre, Toronto, Ontario M4N 3M5, Canada; 2 Department of Immunology, University of Toronto, Toronto, Ontario M5S 1A1, Canada

**Keywords:** browning, burn injury, hypermetabolism, IL6, lipolysis, NLRP3

A severe burn injury is one of the most devastating injuries and is associated with a high risk of mortality (~180,000 deaths per year worldwide) [1]. In addition to large wounds, severe burns can result in injury-induced hypermetabolism, which is a major challenge faced by clinicians in the treatment of patients [2]. Hypermetabolism is known to trigger a plethora of metabolic disorders including type 2 diabetic milieu, fatty liver, sepsis and multiple organ failure [2,3]. As a matter of fact, hypermetabolism can persist for years, consistently putting a high demand of energy on the body, thus making it extremely difficult for the metabolic system to meet the body’s existing nutritional requirements [2]. The hypermetabolic response in burn patients can be triggered by a wide variety of signaling mediators such as catecholamines, glucocorticoids, cytokines or other inflammatory signaling mediators, making it very heterogeneous in nature [4]. Given the challenges induced by hypermetabolism in burn patients, there is a growing need to understand and modulate hypermetabolism in burn patients to improve their clinical outcomes.

Increasing evidence suggests that the hypermetabolic response is partly fueled by the fat mass depots or the white adipose tissue (WAT) in burn patients [5]. The burn-induced hypermetabolic response activates a cascade of events in WAT that fuels the long-lasting hypermetabolic response in burn patients. In hypermetabolic conditions, WAT undergoes lipolysis (breaking down fats) and initiate a browning response (expending fat to heat production) to meet the increased energy demand in burn patients [5]. WAT acquires the evolutionarily conserved characteristics of brown adipose tissue (i.e., enhanced mitochondrial uncoupling and biogenesis) that switches the indigenous characteristics of WAT from fat storage to energy expenditure. This phenomenon is referred to as ‘browning,’ which causes nonshivering thermogenesis or heat production [5,6], resulting in increased resting energy expenditure (REE) that perturbs the energy balance in burn patients [5]. WAT browning in burn patients was first acknowledged in 2015 when two independent research groups reported that severe burn injury induces WAT browning and significantly contributes to the hypermetabolic state in mice and humans [7,8]. Furthermore, Patsouris *et al.* delineated the role of catecholamines and *IL-6* in triggering WAT browning after burn injury [7]. In addition to this, several key metabolic markers were identified as being altered in early versus late phase WAT of burn patients [8], which is still not understood. Nevertheless, these two hallmark studies claimed the association of WAT browning to the hypermetabolic response in burn patients, suggesting the dire need for the high throughput screening of biochemical pathways and therapeutic interventions to prevent or attenuate WAT browning and the associated feed-forward hypermetabolic response. To have more detailed information on the role of WAT browning in burn-associated hypermetabolism, Abdullahi *et al.* provides a detailed review in this context [5].

The phenomenon of triggering WAT browning was further delineated by following a lead on the plausible role of *IL-6* and other inflammatory mediators acting as ‘messengers’ to activate the nonshivering thermogenesis in hypermetabolic conditions [9]. It is well established that the sympathetic nervous system transcends messages to activate WAT browning by releasing catecholamines and other signaling mediators, which activate nonshivering thermogenesis [10]. This hypothesis was tested by challenging *IL-6*-deficient mice with burn injury. Indeed, *IL-6*-deficient mice demonstrated a protective response against the induction of WAT browning and had better metabolic outcomes after burn injury [9]. Also, studies in *IL-6*-deficient mice demonstrated the association of enhanced infiltration and migration of type 2 macrophages in WAT after burn injury, implicating the crucial role of *IL-6* in WAT browning [11]. Although targeting *IL-6* has reduced macrophage infiltration and reduced WAT browning after burn injury in a murine model [11], the mechanisms underpinning the role of macrophages and inflammatory mediators in initiating the hypermetabolic response are not fully understood. For instance, targeting the critical inflammasome mediator *NLRP3* inflammasome after burn injury has demonstrated a crucial need of *NLRP3* in WAT remodeling and mitochondrial function [12]. *NLRP3* is a multifactor protein complex, which plays a pivotal role in regulating macrophage differentiation and inflammatory response to infections and helps in wound healing [13]. Notably, *NLRP3* was found activated in the early phase in burn patients. However, when challenged with burn injury, *NLRP3*-deficient mice had augmented WAT browning response [12] and enhanced hepatic fatty liver development [14], indicating the protective role of *NLRP3* in WAT remodeling. These studies demonstrate that although macrophages and inflammatory mediators were considered detrimental in initiating WAT browning, *NLRP3* murine studies have challenged the notion, warranting the need for detailed mechanistic studies to avoid potential side-effects of future targets. Furthermore, murine studies suggest that *NLRP3* has an apparent role in eliciting reactive oxygen species production and is protective in attenuating WAT browning and mitochondrial dysfunction [15]. However, the exact metabolic role of *NLRP3* and reactive oxygen species signaling in the burn-induced hypermetabolic response has yet to be elucidated.

In hypermetabolic conditions, WAT browning (or wasting) not only leads to a decline in body mass reserves, it also substantially enhances the amount of circulating free fatty acids due to WAT lipolysis or breakdown [4,5]. Beyond the role of WAT browning in fueling hypermetabolism, rise in systemic fat circulation leads to ectopic fat deposition in vital organs such as heart, kidney and liver, which affects burn patients survival outcomes [4,5]. Given the importance of WAT browning in fueling the hypermetabolism and metabolic dysfunction post-burn injury, it was deemed important to understand the potential ways to reduce WAT browning and associated lipolysis. Several hypotheses were tested and assessed to address this clinical challenge on how to reduce WAT browning and their eventual consequences to the hypermetabolic microenvironment. The most promising drugs that were effective in reducing WAT browning and lipolysis in hypermetabolic conditions include propranolol [7], metformin [16] and acipimox [17]. Propranolol is a nonselective β-1/2 receptor antagonist widely used to treat burn patients [5,6]. Clinical studies assessing propranolol have shown benefits in reducing REE and muscle catabolism in patients [18,19]. Also, propranolol treatment resulted in reducing the expression of browning associated metabolic markers in WAT obtained from burn patients, possibly by blocking adipocytic β-1/2 receptors [7]. However, the tissue-specific benefits of this drug are yet to be elucidated. Another school of thought includes assessing the role of an antidiabetic drug metformin which is generally used to control hyperglycemic conditions in burn-associated hypermetabolic conditions. Clinical studies assessing the efficacy of metformin (an antidiabetic drug) in burn patients have demonstrated its benefits in reducing plasma blood glucose levels and reducing muscle catabolism [20]. In addition to this, metformin has also shown beneficial effects in controlling blood glucose levels in a Phase II randomized controlled trial in patients with over 20% total burn surface area [21]. Interestingly, mechanistic studies elucidating the action of metformin in a murine model revealed its benefits associated with decreasing WAT browning and mitochondrial oxidation, while preserving the WAT mass by activating protein phosphatase 2A [16], suggesting a protective ‘whitening’ effect of metformin against severe burn injury. Another hypothesis assessed was the possible effect of a niacin derivative known as acipimox, which has shown promising results in reducing circulating fat content and improved insulin sensitivity in obese patients [22]. Acipimox specifically targets protein kinase A mediated lipolysis by reducing the substrate content [23]. When administered in mice challenged with severe burn injury, acipimox has rendered its protective effect by reducing WAT lipolysis and browning [17]. Acipimox is also effective in lowering systemic fat circulation and hepatic fat infiltration after severe burn injury in a murine model [17]. However, the mechanistic action, safety and efficacy of this drug in burn patients are yet to be assessed.

Collectively, these studies demonstrate that targeting WAT (or fat) browning is promising yet challenging and complex, as this process drives lipotoxicity when inflicted with burns but also promotes insulin sensitivity in metabolic disorders such as obesity and diabetes. Nevertheless, selective targeting of WAT browning seems to be a promising therapeutic intervention in burn patients to combat the hypermetabolic response after burn injury. Successful clinical drugs include propranolol and metformin, which have shown positive results in burn patients. However, only metformin has shown promising tissue-specific effects in murine studies. In the future, detailed clinical research is required to unveil the specificity, safety and efficacy of these drugs in reducing the WAT browning and associated hypermetabolic response in burn patients. Moreover, the exact mechanistic action of catecholamines, glucocorticoids and other signaling mediators in initiating WAT browning after burn injury is still not understood. Overall, this work highlights the mechanistic and clinical advancement achieved thus far in targeting WAT browning to curtail the hypermetabolic response, which is equally as challenging and important as unlocking its thermogenic potential in reducing the obesity epidemic.
